# Whole-exome sequencing prioritizes candidate genes for hereditary cataract in the Emory mouse mutant

**DOI:** 10.1093/g3journal/jkad055

**Published:** 2023-03-09

**Authors:** Thomas M Bennett, Yuefang Zhou, Kacie J Meyer, Michael G Anderson, Alan Shiels

**Affiliations:** Department of Ophthalmology and Visual Sciences, Washington University School of Medicine, St. Louis, MO 63110, USA; Department of Ophthalmology and Visual Sciences, Washington University School of Medicine, St. Louis, MO 63110, USA; Department of Molecular Physiology and Biophysics, Carver College of Medicine, University of Iowa, Iowa City, IA 52242, USA; Department of Molecular Physiology and Biophysics, Carver College of Medicine, University of Iowa, Iowa City, IA 52242, USA; Department of Ophthalmology and Visual Sciences, Washington University School of Medicine, St. Louis, MO 63110, USA

**Keywords:** lens, cataract, CFW-*Em*/J mouse, *Prx*, *Adamts10*, *Abhd12*

## Abstract

The Emory cataract (*Em*) mouse mutant has long been proposed as an animal model for age-related or senile cataract in humans—a leading cause of visual impairment. However, the genetic defect(s) underlying the autosomal dominant *Em* phenotype remains elusive. Here, we confirmed development of the cataract phenotype in commercially available *Em*/J mice [but not ancestral Carworth Farms White (CFW) mice] at 6–8 months of age and undertook whole-exome sequencing of candidate genes for *Em*. Analysis of coding and splice-site variants did not identify any disease-causing/associated mutations in over 450 genes known to underlie inherited and age-related forms of cataract and other lens disorders in humans and mice, including genes for lens crystallins, membrane/cytoskeleton proteins, DNA/RNA-binding proteins, and those associated with syndromic/systemic forms of cataract. However, we identified three cataract/lens-associated genes each with one novel homozygous variant including predicted missense substitutions in *Prx* (p.R167C) and *Adamts10* (p.P761L) and a disruptive in-frame deletion variant (predicted missense) in *Abhd12* (p.L30_A32delinsS) that were absent in CFW and over 35 other mouse strains. *In silico* analysis predicted that the missense substitutions in *Prx* and *Adamts10* were borderline neutral/damaging and neutral, respectively, at the protein function level, whereas, that in *Abhd12* was functionally damaging. Both the human counterparts of *Adamts10* and *Abhd12* are clinically associated with syndromic forms of cataract known as Weil-Marchesani syndrome 1 and polyneuropathy, hearing loss, ataxia, retinitis pigmentosa, and cataract syndrome, respectively. Overall, while we cannot exclude *Prx* and *Adamts10*, our data suggest that *Abhd12* is a promising candidate gene for cataract in the *Em*/J mouse.

## Introduction

Clouding of the crystalline lens, or cataract(s), is commonly associated with aging and, despite advances in surgical treatment, age-related or senile cataract is a leading cause of visual impairment (low vision and blindness) worldwide (GBD 2019 Blindness and Vision Impairment Collaborators 2021). Cataract may also be inherited either as an isolated phenotype or as part of a multisystem disease typically with an early-onset (birth to 40 years), and over 450 underlying genes have been identified including those for lens crystallins, connexins and other membrane proteins, ocular transcription factors, and RNA-binding proteins (Shiels and Hejtmancik 2017, 2019, 2021) (https://cat-map.wustl.edu). Hereditary forms of cataract also afflict many domesticated animals including sheep, cattle, horses/ponies, and over 60 breeds of dogs (Pinard and Basrur 2011; Wilson *et al*. 2012; Mellersh 2014; Murgiano *et al*. 2014; Ricketts *et al*. 2015). In addition, numerous inbred strains of laboratory rats and mice serve as animal models of human cataract including the Shumiya cataract rat and senescence-accelerated mouse strains (Mori *et al*. 2006; Graw 2019).

The Emory cataract (Gene symbol: *Em*) mutation arose spontaneously in an inbred colony of Carworth Farms White (CFW) mice, and due to the relatively late onset of autosomal dominant lens opacities at 6–8 months of age, the *Em* mouse was originally proposed (in 1981) as an animal model for human senile cataract (Kuck *et al*. 1981; Kuck 1990). In vivo imaging of the *Em* mouse lens revealed that the cataract progressed slowly with variable severity in four stages starting with irregularly round opacification confined to the central region of the anterior superficial cortex by 2 months of age (stage-1), progressing into the anterior deep cortex by 6 months (stage-2), then to the supranuclear region by 7–8 months (stage-3), and eventually to total lens opacification by 10–12 months (stage-4) (Takizawa and Sasaki 1986). Histopathological studies of *Em* lenses using light and electron microscopy have detected early ultrastructural changes including acellular regions in the anterior epithelium by 2 weeks of age and swelling of the anterior cortical fibers by 1–2 months of age (Uga *et al*. 1988). Cataract onset was delayed in *Em* mice fed a calorie (20–40%) restricted diet (Taylor *et al*. 1989, 1995a, 1995b; Scrofano *et al*. 1998a, 1998b), and cataract grading studies have shown that beyond 6 months of age, *Em* females develop cataract more rapidly (7–11 months) than age-matched males, whereas, the final cataract severity (≥13 months) was indistinguishable between sexes (Shang e*t al.* 2002).

Biochemical studies of *Em* lenses have found a range of abnormalities including increased levels of protein insolubility, Ca^2+^ ions, oxidized glutathione, and membrane lipid peroxidation (Kuck 1990). Lens gene expression studies have detected down-regulation of several mRNA transcripts for crystallins (e.g. *Cryaa*) and major intrinsic protein or aquaporin-0 (MIP/AQP0), and up-regulation of adhesion related kinase (Ark) receptor tyrosine kinase associated with cataract in the *Em* lens (Shi and Bekhor 1992; Sheets *et al*. 2002). Further, ultrastructural and immunochemical studies have shown that cortical cataract formation in the *Em* lens was associated with premature proteolytic-cleavage of MIP/AQP0 and gap-junction alpha-8 protein or connexin-50 (GJA8/Cx50) C-termini resulting in abnormal wavy square-array junctions and smaller gap-junctions, respectively, compared to wild-type lens fiber cells (Biswas *et al*. 2014). Finally, imbalances in the lens crystallin proteome and changes in transfer RNA-derived fragments have been associated with cataract development in the *Em* mouse (Schmid *et al*. 2021; Zhang *et al*. 2022). However, all of these observed changes are likely secondary to the underlying genetic defect(s). Here we have undertaken a whole-exome sequencing approach to analyze variants in candidate genes for *Em*.

## Materials and methods

### Mice

CFW-*Em*/J (*Em*/J, Stock no. 001998) and C57BL/6J (B6J, Stock no. 000664) mice were obtained from The Jackson Laboratory (Bar Harbor, ME, USA). CFW(SW) mice (Strain code 024) were obtained from Charles River Laboratories (Wilmington, MA, USA). Lens imaging of conscious mice was performed using a slit-lamp at 25× magnifications (SL-D7; Topcon, Tokyo, Japan) equipped with a digital camera (D800; Nikon, Tokyo, Japan) under identical image acquisition and processing settings. Anterior chamber imaging of mice was performed by spectral domain-ocular coherence tomography using a 12-mm bore (Bioptigen Envisu, Leica Microsystems, Deerfield, IL, USA). Briefly, mice were anesthetized with ketamine (100 mg/Kg body-weight) and xylazine (10 mg/Kg body-weight) and both eyes immediately hydrated with balanced salt solution (BSS, Alcon Laboratories, Fort Worth, TX, USA). Radial volume scan parameters within the Bioptigen InVivoVueClinic software were set to: 2-mm diameter, 1,000 A-scans/B-scan, 100 B-scans/volume, 1 frame/B-scan, and 1 volume. Central corneal thickness was measured within the Bioptigen InVivoVueClinic Sofware using vertical angle-locked B-scan calipers. Mice were humanely euthanized according to the American Veterinary Medical Association and the eyes were removed. Whole globes or dissected lenses immersed in phosphate buffered saline (#P4417-100TAB, MilliporeSigma, Burlington, MA, USA) were imaged with a dissecting microscope (Stemi 2000, Zeiss, Thornwood, NY, USA) equipped with a digital camera (Spot Insight, Sterling Heights, MI, USA). All mouse studies were approved by the Institutional Animal Care and Use Committee at Washington University in St. Louis and the University of Iowa in compliance with the Institute for Laboratory Animal Research guidelines.

### Exome sequencing and variant analysis

Genomic DNA was isolated from mouse spleen using the Gentra Puregene Kit (Qiagen, Valencia, CA, USA) and quantified by absorbance at 260 nm (NanoDrop 2000, Wilmington, DE, USA). Whole exome capture was achieved using the SureSelect Mouse All Exon (50 Mb) Kit that targets over 221,784 exons from 24,306 genes (Agilent Technologies, Santa Clara, CA, USA) followed by next-generation sequencing (1 lane, paired-end reads 2 × 101 bps) on an Illumina HiSeq 2000 system using the Multiplexing Sample Preparation Oligo-nucleotide Kit and the HiSeq 2000 Paired-End Cluster Generation Kit (Illumina, San Diego, CA, USA) according to the manufacturers’ instructions and as briefly described (Mackay *et al*. 2014, 2015). Raw sequence data were aligned to the mouse reference genome (build mm10) by NovoalignMPI (www.novocraft.com), and sequence variants were called using the Sequence Alignment/Map format (SAMtools) and Picard programs (http://samtools.sourceforge.net/). Target coverage and read-depth were reviewed by the Integrated Genomics Viewer (IGV; http://www.broadinstitute.org/igv/). Called variants were reviewed using the SNP and Variation Suite software (SVS 8.9.1, Golden Helix, Bozeman, MT, USA). Variant effects on protein function were predicted using the Sorting Intolerant from Tolerant (SIFT) web server (https://sift.bii.a-star.edu.sg/) (Sim *et al*. 2012) and the Protein Variation Effect Analyzer (PROVEAN) web server (http://provean.jcvi.org) (Choi and Chan 2015).

### Polymerase chain reaction amplification and sequencing

Allele-specific polymerase chain reaction (PCR) amplification was performed in a GeneAmp 9700 thermal cycler (Applied Biosystems, Grand Island, NY, USA) using Top Taq mastermix kit (Qiagen, Valencia, CA, USA) and three (forward, reverse, nested) gene-specific primers (Supplementary Table 1) followed by horizontal agarose-gel electrophoresis (BioRad, Hercules, CA, USA) with GelRed (Biotium, Hayward, CA, USA) as described (Shiels *et al*. 2008). PCR-Sanger sequencing was performed in both directions using M13-tailed gene-specific primers (Supplementary Table 1) with the BigDye Terminator v3.1 kit and a 3130xl Genetic Analyzer (Applied Biosystems) as described (Mackay *et al*. 2014, 2015).

## Results

### *Em*/J mouse lens phenotype

**Fig. 1.**
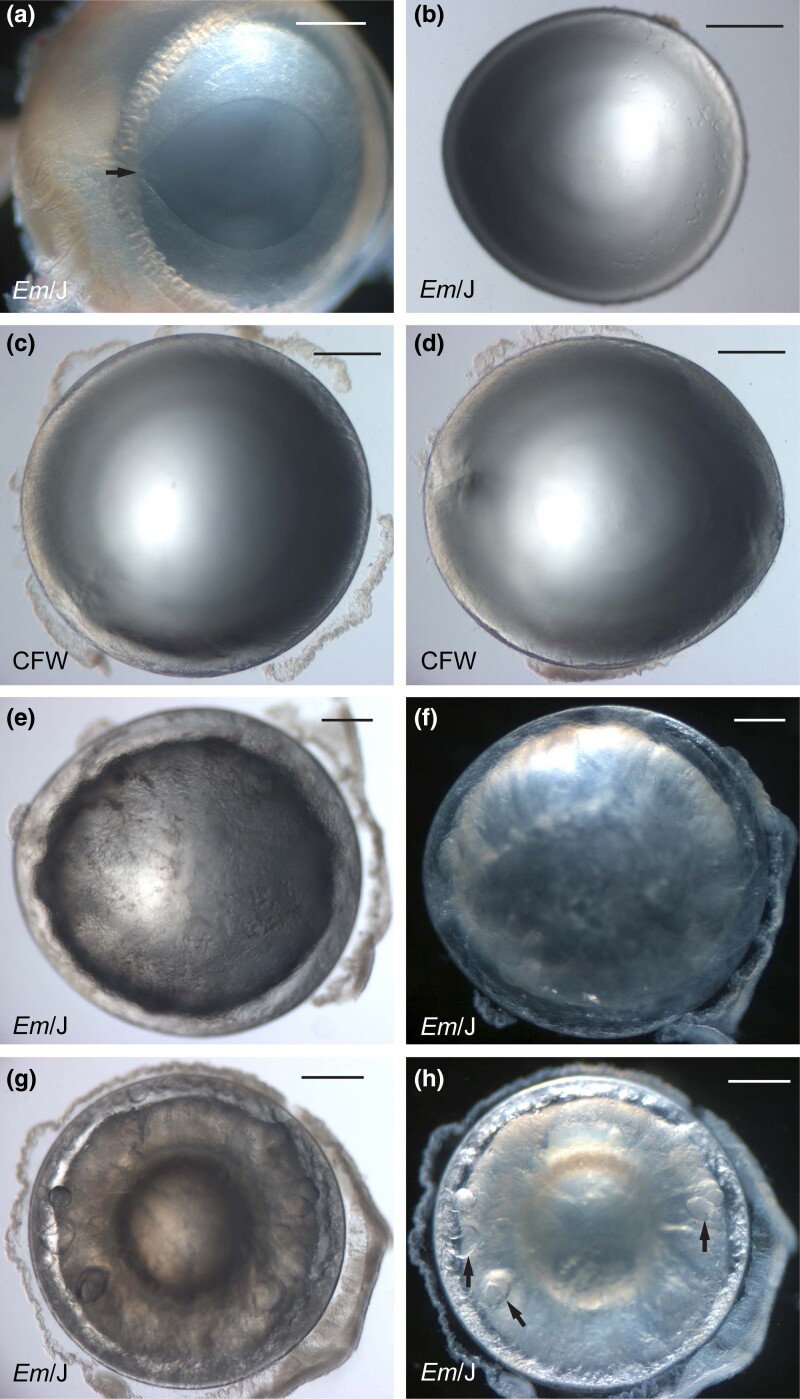
Lens phenotype of *Em*/J mice. Dark-field (a, f, h) and bright-field (b–d, e, g) dissecting microscope images of *Em*/J eye (a) and lenses (b, e–h) and CFW lenses (c, d) at P21 (a, b), 7 months of age (c–f), and 9 months of age (g, h). Arrow in (a) indicates pupil displacement. Arrows in (h) indicate signs of liquefaction. Scale bar: 500 µm.

**Fig. 2.**
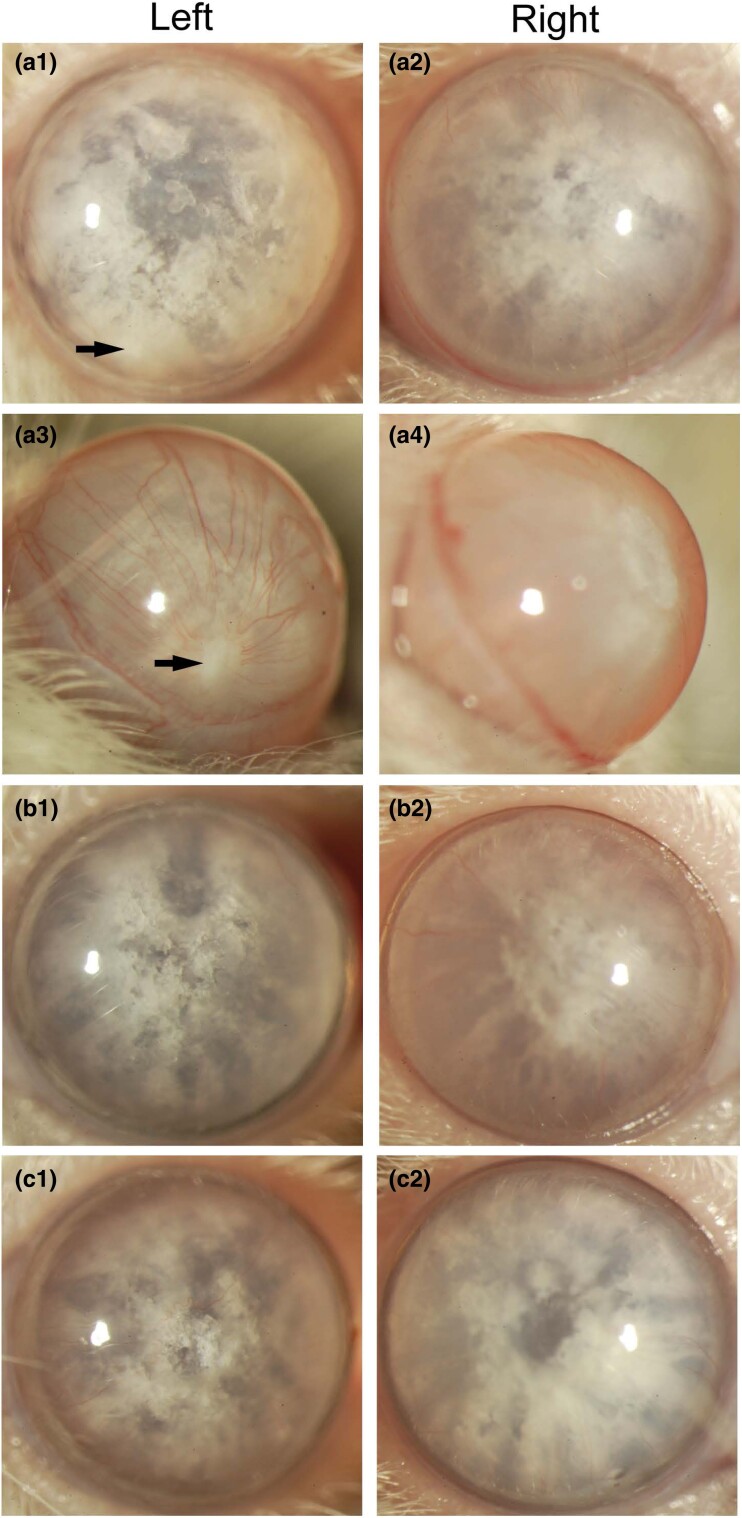
Slit-lamp images (25× magnification) of left and right eyes from three *Em*/J mice at 7 months of age (a–c). Arrows in A1 and A3 indicate a suspected synechia associated with pupil displacement.

*Em*/J inbred albino mice are reported to be homozygous for an autosomal dominant cataract (*Em*) phenotype that arose spontaneously in an inbred colony of CFW mice at Emory University, Atlanta, GA, USA (Kuck *et al*. 1981; Kuck 1990) (https://www.jax.org). CFW mice were derived from so-called Swiss mice and have been maintained as an outbred population for many generations causing reduced linkage disequilibrium between nearby alleles/markers compared to inbred mouse strains—rendering them useful for genetic analysis of complex traits (Lynch 1969; Chia *et al*. 2005; Yalcin *et al*. 2010; Parker *et al*. 2016). First, we performed a gross comparison of postmortem eyes and lenses from *Em*/J (mutant sub-strain) and CFW (ancestral strain) mice (n = ≥3 animals) before and after obvious cataract formation in the former. While *Em*/J and CFW eyes were grossly indistinguishable at post-natal day 21 (P21), we observed occasional, unilateral, displacement of the pupil margin in both strains (Fig. 1a). Upon dissection, the otherwise clear lenses from pupil-displaced *Em*/J eyes were found to be misshapen with an “egg-shaped” appearance (Fig. 1b). Similarly, at 7 months of age, CFW lenses were transparent with an occasional, unilateral, egg-shaped appearance (Fig. 1c and d). These observations suggested that variable pupil displacement may be due to an adhesion between the iris and lens capsule (suspected posterior synechia) that resulted in asymmetric lens growth. Regardless, occasional pupil displacement with a misshapen lens in the *Em*/J mutant sub-strain was likely derived from the ancestral CFW strain. In *Em*/J lenses at 7 months of age, severe, bilateral, cortical, cataract was manifest without signs of lens rupture (Fig. 1e and f) and by 9 months of age, *Em*/J lenses appeared slightly shrunken with signs of liquefaction in the outer cortex region surrounding the nucleus (Fig. 1g and h). Slit-lamp examination of conscious *Em*/J mice (without pupil dilation or anesthesia) at 7 months of age confirmed the presence of bilateral, cortical cataract with mild inter-ocular variability and, in one case, a suspected synechia without other signs of ocular inflammation (Fig. 2a1 and a3). Otherwise, *Em*/J (and CFW) eyes had clear corneas and translucent irises with unremarkable central corneal thickness (average CCT = 99.4 µm ± SD 2.51, n = 6 eyes) and anterior chamber dimensions (Supplementary Fig. 1) (Lively *et al*. 2010).

Subsequently, in an effort to genetically map the *Em* phenotype to a mouse chromosome, we began an F2 intercross with the C57BL/6J (B6J) genome reference strain (Sarsani *et al*. 2019). However, F1 progeny failed to develop the characteristic cataract phenotype by 9–12 months of age suggesting that heterozygous *Em* mice developed cataract much later than homozygotes and/or that the cataract exhibited reduced penetrance on the B6J genetic background—making further genetic mapping of *Em* challenging.

### Exome metrics and variant analysis

For both *Em*/J and CFW exome samples, over 86% of total paired-end reads were mapped to the C57BL/6J mouse reference genome assembly (Genome Reference Consortium Mouse Build 38 or GRCm38/mm10) and over 86% of mapped reads were present in the captured exome sequences (Supplementary Table 2). Over 98% of each exome achieved a read depth of ≥10 × coverage and over 93% of each achieved 25× coverage. Unexpected gaps in coverage were found on chromosome-4 of CFW (32 exons) and on chromosome Y of *Em*/J (140 exons). However, close inspection of these regions of low coverage using the IGV (https://software.broadinstitute.org/software/igv/) did not reveal credible candidate genes for ocular abnormalities.

Exome variant call files were filtered against the National Center for Biotechnology Information RefSeq genes 59 and dbSNP 146 public databases using the SNP and Variation Suite (SVS) software package version 8.9.1 (https://www.goldenhelix.com). SVS generated over 87,000 variants that were subdivided into 13 sub-types, including intron > synonymous > missense > splice-site > untranslated region (UTR) > non-coding exon > upstream gene > downstream gene > in-frame insertions/deletions (indels) > frameshift > stop-codon > initiator (start) codon > disruptive in-frame indels in descending order of abundance (Table 1 and Supplementary Table 3). We excluded intron variants, synonymous variants, upstream and downstream intergenic variants, non-coding variants, and most UTR variants—accounting for over 80% of the total filtered variants (Table 1). Of the remainder, we focused on (1) predicted missense variants including single nucleotide variants (SNVs) resulting in amino-acid substitutions, in-frame indels, and disruptive in-frame indels and (2) predicted loss-of-function (LoF) variants including splice-acceptor/donor variants, frameshift variants, stop-gain/loss variants, and start-codon variants (Supplementary Table 3). Of these missense (∼15,000) and LoF (∼419) variants, the vast majority (>90%) had reference SNP (rs) identifiers effectively excluding them as candidate genes for *Em* (Supplementary Table 3).

**Table 1.** Summary of exome variants found in *Em*/J and CFW mice.

Variant type	Gene region	Total (%) {subtotal}	Effect (Clinically Relevant)
1. Intron	intron	32,467 (37.26%)	Other
2. Synonymous	exon	28,089 (32.24%)	Other
3. Missense	exon	15,011 (17.23%)	Missense
4. Splice-site		4,614 (5.30%)	
splice-acceptor	intron	{33}	Loss-of-function (LoF)
splice-donor	intron	{26}	Loss-of-function
splice-region	intron	{4556}	Other
5. Untranslated region (UTR)		4,479 (5.14%)	
3′-UTR	UTR3	{3034}	Other
5'-UTR	UTR5	{1445}	Missense, other
6. Non-coding exon	exon	717 (0.82%)	Other
7. Upstream gene	upstream_intergenic	577 (0.66%)	Unknown
8. Downstream gene	downstream_intergenic	453 (0.52%)	Unknown
9. In-frame indel		332 (0.38%)	
inframe_deletion	exon	{177}	Missense, other
inframe_insertion	exon	{155}	Missense, other
10. Frameshift	exon	210 (0.24%)	Loss-of-Function
11. Stop codon		147 (0.17%)	
stop_gained (nonsense)	exon	{108}	Loss-of-Function
stop_lost	exon	{20}	Loss-of-Function
stop_retained	exon	{19}	Other
12. Inatiator (start) codon	exon	22 (0.03%)	Loss-of-function
13. Disruptive in-frame indels		20 (0.02%)	
disruptive_inframe_deletion	exon	{9}	Missense
disruptive_inframe_insertion	exon	{11}	Missense
	Total	87,138	

Since *Em*/J (and CFW) mice are albinos with pink eyes, we first sought to confirm the presence of an underlying mutation in the tyrosinase gene (*Tyr*) on chromosome-7 (Beermann *et al*. 2004). IGV confirmed that both strains were homozygous for the classical albino missense mutation (c.G308C, p.C103S) in exon-1 of *Tyr* (Supplementary Table 3). In addition, we found that both *Em*/J and CFW mice were homozygous for a nonsense mutation (p.Y347X) in exon-7 of the gene for phosphodiesterase 6 beta-subunit (*Pde6b*) on chromosome-5 (Supplementary Table 3) that underlies an autosomal recessive form of retinal (rod-photoreceptor) degeneration 1 (rd1, rodless)—consistent with prior reports that CFW mice undergo early-onset retinal degeneration, (Kuck *et al*. 1981; Serfilippi *et al*. 2004; Han *et al*. 2013).

### Variant analysis of known genes for cataract

Our priority was to exclude the possibility that a well-known gene for cataract was causative in the *Em*/J mutant mouse. Currently, over 450 genes across the genome (autosomes, X, and mitochondrial chromosomes) have been associated with inherited and age-related forms of cataract and other lens disorders—with or without other ocular and/or systemic abnormalities—in humans and mice (https://cat-map.wustl.edu, www.omim.org). Such genes include those for lens crystallins (e.g. *Cryaa*, *Cryab*, *Cryba1*, *Cryba2*, *Crybb1*, *Crybb2*, *Cryga-f*, *Crygs*, *Cryz*), transmembrane proteins (e.g. *Gja3, Gja8, Mip/Aqp0, Lim2, Epha2*) membrane-associated proteins (e.g. *Chmp4b*, *Fyco1*, *Efna5*, *Prx*, *Lctl/Klph*), cytoskeletal proteins (*Bfsp1*, *Bfsp2*, *Vim*), transcription factors (e.g. *Hsf4*, *Pitx3*, *Foxe3*, *Pax6*, *Prox1*, *Yap1*), RNA-binding proteins (e.g. *Tdrd7*, *Celf1*), a lysosomal enzyme (*Dnase2b*), and syndromic/systemic forms of cataract (e.g. *Galk1*, *Gcnt2*, *Pxdn*, *Agk*, *Ftl1*, *Phyh*). In addition, several genes that serve important roles in lens cell biology but have not yet been directly associated with cataracts are listed in the Cat-Map database (https://cat-map.wustl.edu). These include genes coding for lens membrane proteins (*Grifin*, *Kl*), a cytoskeleton/chaperone protein (*Lgsn*), organelle degradation proteins (*Bnip3l/Nix*, *Plaat3*, *Cdk1*), and an epithelial cell protein (*Lenep*). First, we matched the Cat-Map gene list against *Em*/J and CFW exome variants with predicted loss-of-function and obtained 1 hit for frameshift variants, 3 hits for stop-gain/loss variants, and 0 hits for splice-donor/acceptor and start-codon variants (Supplementary Table 3). Second, we matched the Cat-Map genes against *Em*/J and CFW exome variants predicted to be missense. We obtained 114 hits with missense SNVs, 6 hits with in-frame indels, and 2 hits with disruptive in-frame indels (Supplementary Table 3). Third, we pooled all the variant hits without rs identifiers in dbSNP 146 to give a list of 43 variants including 39 missense SNVs, 1 stop-gained variant, 1 frameshift variant, 1 in-frame insertion variant, and 1 disruptive in-frame deletion variant found in 26 Cat-Map genes (Table 2).

**Table 2.** Variants in known genes for cataract found by filtering *Em*/J and CFW exome variants against the RefSeq genes 59, dbSNP 146, and ensembl 106 databases.

Marker coordinates	Reference	Alternates	Gene Names	Sequence Ontology (Combined)	Gene Region	Transcript no. and HGVS c. (Clinically Relevant)	HGVS p. (Clinically Relevant)	Sequence Ontology (Clinically Relevant)	Effect (Clinically Relevant)	CFW:Em/J (IGV)	Ensembl 106 (UCSC)	Other mouse strains	Comments
1:34191473-SNV	G	A	*Dst*	missense_variant	exon 39	NM_001276764:c.8680G > A	p.Val2894Ile	missense_variant	Missense	G/A:A/A	rs31675513	CBA,DBA = A/A	
1:65926877-SNV	A	G	*Crygf*	missense_variant	exon 2	NM_027010:c.239A > G	p.His80Arg	missense_variant	Missense	G/G:G/G	rs3666875	129,FVB = G/G	
1:65928211-SNV	G	A	*Crygf*	missense_variant	exon 39	NM_027010:c.493G > A	p.Gly165Ser	missense_variant	Missense	A/A:A/A	rs31714794	129,FVB = A/A	
1:188810274-SNV	A	G	*Ush2a*	missense_variant	exon 50	NM_021408:c.10036A > G	p.Thr3346Ala	missense_variant	Missense	A/A:G/G	rs30740177	129,CBA = G/G	
2:69477073-SNV	T	C	*Lrp2*	missense_variant	exon 46	NM_001081088:c.8641A > G	p.Ser2881Gly	missense_variant	Missense	C/C:T/T			Em/J = reference
2:69486236-SNV	T	C	*Lrp2*	missense_variant	exon 38	NM_001081088:c.6400A > G	p.Asn2134Asp	missense_variant	Missense	C/C:T/T			Em/J = reference
2:69505255-SNV	C	T	*Lrp2*	missense_variant	exon 26	NM_001081088:c.4123G > A	p.Val1375Met	missense_variant	Missense	T/T:C/C		ZALENDE/EiJ = T/T	Em/J = reference
2:118632425-SNV	G	A	*Bub1b*	missense_variant	exon 18	NM_009773:c.2266G > A	p.Val756Ile	missense_variant	Missense	T/T:C/C			Em/J = reference
2:150904454-Del	CGTCCA	-	*Abhd12*	disruptive_inframe_deletion	exon 18	NM_024465:c.89_94delTGGACG	p.Leu30_Ala32delinsPro	disruptive_inframe_deletion	Missense	ref/ref:del + A/del + A			PROVEAN score = −20.74
3:103818891-SNV	A	G	*Ap4b1*	missense_variant	exon 7	NM_026193:c.1286A > G	p.Asn429Ser	missense_variant	Missense	G/G:G/G	rs31738189	129,FVB = G/G	
3:103821362-SNV	G	A	*Ap4b1*	missense_variant	exon 10	NM_026193:c.1819G > A	p.Glu607Lys	missense_variant	Missense	A/A:A/A	rs4224154	129,FVB = A/A	
4:53737612-SNV	T	A	*Fktn*	missense_variant	exon 6	NM_139309:c.733T > A	p.Ser245Thr	missense_variant	Missense	T/T:T/T(A)			Em/J low coverage (2 of 16 reads)
4:137542546-SNV	C	A	*Hspg2*	missense_variant	exon 54	NM_008305:c.6905C > A	p.Ser2302Tyr	missense_variant	Missense	C/A:C/C		ZALENDE/EiJ,I/LnJ = A/A	Em/J = reference
4:148041660-SNV	A	G	*Mthfr*	missense_variant	exon 2	NR_027809:n.210A > G, NM_010840:c.187A > G	p.Ser63Gly	missense_variant	Missense	G/G:G/G	rs27617540	NOD/SPRET = G/G	
4:148043548-SNV	C	A	*Mthfr*	stop_gained	exon 3	NR_027809:n.503C > A, NM_010840:c.357C > A	p.Cys119*	stop gained_variant	LoF	C/C(A):C/C			Em/J = reference; CFW low coverage (2 of 18 reads)
4:148929159-SNV	A	G	*Casz1*	missense_variant	exon 5	NM_001159344:c.179A > G	p.His60Arg	missense_variant	Missense	G/G:G/G	rs231173480	129,CBA = G/G	
4:148929467-SNV	A	G	*Casz1*	missense_variant	exon 5	NM_001159344:c.487A > G	p.Thr163Ala	missense_variant	Missense	G/G:G/G	rs250799899	129,CBA = G/G	
4:148938614-SNV	A	G	*Casz1*	missense_variant	exon 11	NM_001159344:c.1975A > G	p.Met659Val	missense_variant	Missense	G/G:G/G	rs32565905	129,CBA = G/G	
4:148939231-SNV	T	G	*Casz1*	missense_variant	exon 11	NM_001159344:c.2592T > G	p.Ile864Met	missense_variant	Missense	G/G:G/G	rs32566624	129,CBA = G/G	
4:148942940-SNV	G	A	*Casz1*	missense_variant	exon 14	NM_001159344:c.2918G > A	p.Ser973Asn	missense_variant	Missense	A/A:A/A	rs51947548	129,CBA = A/A	
4:148944359-SNV	T	C	*Casz1*	missense_variant	exon 16	NM_001159344:c.3260T > C	p.Leu1087Pro	missense_variant	Missense	C/C:C/C	rs49392164	All strains = C/C	
5:43699942-SNV	G	T	*Cc2d2a*	missense_variant	exon 15	NM_172274:c.1470G > T	p.Gln490His	missense_variant	Missense	G/G(T):G/G			Em/J = reference; CFW low coverage (2 of 15 reads)
5:123511373-SNV	T	G	*B3gnt4,Diablo*	missense_variant	exon 1,6	NM_198611:c.800T > G,NM_023232:c.*1138A > C	p.Met267Arg,?	missense_variant,3_prime_UTR_variant	Missense,Other	G/G:G/G	rs33118476	129,BALBc = G/G	
6:91515726-SNV	C	T	*Xpc*	missense_variant	exon 1	NM_009531:c.74G > A	p.Arg25Gln	missense_variant	Missense	C/C:T/T		KK/HlJ = T/T	
7:27516157-SNV	C	T	*Prx*	missense_variant	exon 7	NM_198048:c.499C > T	p.Arg167Cys	missense_variant	Missense	C/T:T/T			PROVEAN score = −2.31
7:27519613-SNV	G	A	*Prx*	missense_variant	exon 7	NM_198048:c.3955G > A	p.Val1319Ile	missense_variant	Missense	G/A:A/A		I/LnJ = A/A	
7:68226170-Ins	-	GGAGCTGGAGAT	*Igf1r*	inframe_insertion	exon 21	NM_010513:c.3884_3895dupTGGAGATGGAGC	p.Leu1295_Glu1298dup	inframe_insertion	Missense	ins/ins:ins/ins	rs213031273	CAST,SPRET = ins/ins	
7:98076096-SNV	C	A	*Myo7a*	missense_variant	exon 26	NM_001256081:c.3357G > T	p.Lys1119Asn	missense_variant	Missense	C/A:C/C			Em/J = reference
9:49107163-SNV	C	T	*Tmprss5*	missense_variant	exon 4	NM_030709:c.262C > T	p.Arg88Cys	missense_variant	Missense	C/C:T/T		KK/HlJ,MOLF/EiJ = T/T	
9:49114532-SNV	C	T	*Tmprss5*	missense_variant	exon 10	NM_030709:c.973C > T	p.His325Tyr	missense_variant	Missense	C/C:T/T		KK/HlJ,MOLF = T/T	
9:53460911-Ins	-	G	*Atm*	frameshift_variant	exon 46	NM_007499:c.6579dupC	p.Ser2194Glnfs*16	frameshift_variant	LoF	ref/ins:ref/ref			Em/J = reference
9:108489778-SNV	T	G	*Lamb2*	missense_variant	exon 31	NM_008483:c.4928T > G	p.Val1643Gly	missense_variant	Missense	T/G:T/T			Em/J = reference
10:77057379-SNV	G	A	*Col18a1*	missense_variant	exon 37	NM_001109991:c.3797C > T	p.Pro1266Leu	missense_variant	Missense	G/G:A/A		BUB = A/A	
11:76210906-SNV	T	C	*Gemin4*	missense_variant	exon 2	NM_177367:c.3028A > G	p.Thr1010Ala	missense_variant	Missense	T/T:T/C	rs1133566737	C3H,CBA = T/C	
11:76211485-SNV	C	T	*Gemin4*	missense_variant	exon 2	NM_177367:c.2449G > A	p.Ala817Thr	missense_variant	Missense	C/C:C/T		C3H,CBA = C/T	
11:76211500-SNV	C	T	*Gemin4*	missense_variant	exon 2	NM_177367:c.2434G > A	p.Gly812Ser	missense_variant	Missense	C/C:C/T		C3H,CBA = C/T	
12:44566381-SNV	T	G	*Nrcam*	missense_variant	exon 17	NM_176930:c.1854T > G	p.Ser618Arg	missense_variant	Missense	T/G:T/T		ZALENDE/EiJ = G/G	Em/J = reference
12:84829279-SNV	C	T	*Ltbp2*	missense_variant	exon 7	NM_013589:c.1553G > A	p.Arg518His	missense_variant	Missense	C/T:C/C		ST = T/T	Em/J = reference
17:33549039-SNV	C	T	*Adamts10*	missense_variant	exon 19	NR_037707:n.2432C > T, NM_172619:c.2282C > T	p.P761L	missense_variant	Missense	C/T:T/T			PROVEAN score = 0.96
17:34931437-SNV	G	C	*Neu1*	missense_variant	exon 1	NM_010893:c.31G > C	p.Gly11Arg	missense_variant	Missense	G/G:C/C	rs239331144	NOD,PWK = C/C	
17:34931450-SNV	A	G	*Neu1*	missense_variant	exon 1	NM_010893:c.44A > G	p.Tyr15Cys	missense_variant	Missense	A/A:G/G	rs108210643	NOD,SPRET = G/G	
17:34931456-SNV	C	T	*Neu1*	missense_variant	exon 1	NM_010893:c.5°C > T	p.Ala17Val	missense_variant	Missense	C/C:T/T	rs217792506	NOD,SPRET = T/T	
17:34931461-SNV	C	T	*Neu1*	missense_variant	exon 1	NM_010893:c.55C > T	p.Arg19Cys	missense_variant	Missense	C/C:T/T	rs238145843	NOD,SPRET = T/T	

**Fig. 3.**
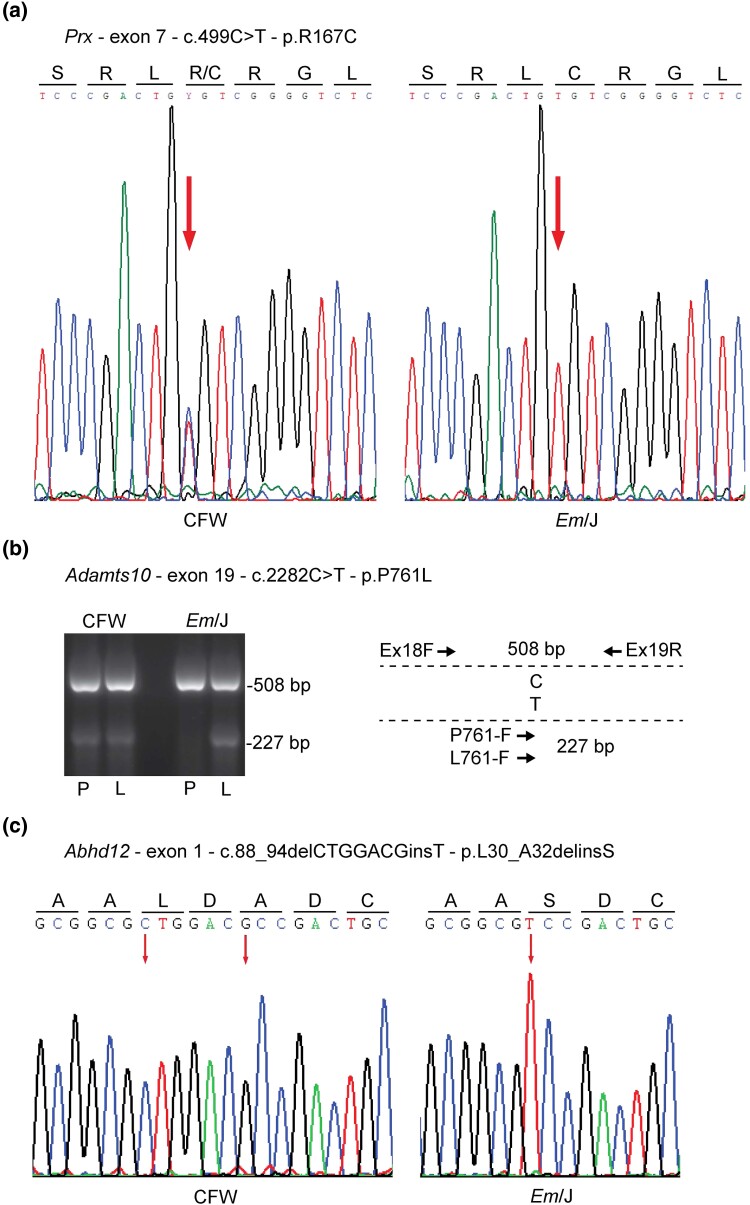
Validation of novel variants in *Prx*, *Adamts10*, and *Abhd12*. a) PCR-Sanger sequencing of exon-7 from *Prx* confirming that CFW and Em/J mice were heterozygous and homozygous for the p.R167C substitution, respectively. b) Allele-specific PCR amplification of exon-19 from *Adamts10* with three primers (Supplementary Table 1), indicated by arrows in the adjacent schematic, followed by gel-electrophoresis confirming that CFW and *Em*/J mice were heterozygous and homozygous, respectively, for the p.P761L substitution. c) PCR-Sanger sequencing of exon-1 from *Abhd12* confirming that *Em*/J mice were homozygous for a p.L30_A32delinsS substitution that was not present in CFW mice.

Fourth, we proceeded to filter these “novel” variants in Cat-Map genes against other public databases including the University of California, Santa Cruz (UCSC) Genome Browser (Lee *et al*. 2022) and the Ensembl Genome Browser (https://ensembl.org) (Cunningham *et al*. 2022) in order to access later versions of dbSNP and genomic variants in other mouse strains. Variant co-ordinates were input to the UCSC Genome Browser and analyzed using the Annotated SNPs from Mouse Strain Comparison Analysis followed by the Ensemble Browser (release 106). Of the 43 first-round variants, 20 were found to have rs identifiers and occurred in other mouse strains (Table 2). A further 19 variants were present in other mouse strains and/or were reference sequence in *Em*-J, leaving four Cat-Map genes each with a novel variant comprising predicted missense SNVs in *Fktn*, *Prx*, and *Adamts10*, and a disruptive in-frame deletion variant in *Abhd12* (Table 2). In order to validate these novel variants, we undertook PCR-Sanger sequencing and/or allele-specific PCR amplification. The *Prx* and *Adamts10* missense SNVs were confirmed to be heterozygous in CFW and homozygous in *Em*/J mice and both were consistent with predicted p.R167C and p.P761L amino-acid substitutions, respectively (Fig. 3a and b). However, the predicted missense substitution (p.S245T) in *Fktn* failed validation in CFW and *Em*/J mice (Supplementary Fig. 2). Close inspection of this variant using IGV revealed that it exhibited very low coverage (12.5% of reads) further suggesting that it was a sequencing artefact. Additionally, we found that the disruptive in-frame 6-bp deletion (c.89_94delTGGACG) in *Abhd12*, predicted to result in a missense substitution (p.L30-A32delinsP), was discordant with PCR-Sanger sequencing. Figure 3c revealed that while the *Abhd12* variant was absent in CFW mice and homozygous in *Em*/J mice, it was more consistent with a 7-bp deletion and 1-bp insertion (c. 88_94delCTGGACGinsT) predicted to result in the missense substitution p.L30_A32delinsS. Alternatively, we cannot exclude the possibility that a 5-bp deletion (c.90_94delGGACG) and a 1-bp deletion (c.88delC), or some other rearrangement, occurred to generate the p.L30_A32delinsS substitution.

Fifth, we used the SIFT web server (https://sift.bii.a-star.edu.sg/) to predict the effects of novel missense SNVs on protein function (Sim *et al*. 2012). A SIFT score of <0.05 was predicted to be intolerant, whereas, a score of ≥0.05 was predicted to be tolerated. The *Prx* substitution (p.R167C) was predicted to be borderline intolerant (score = 0.05), whereas, the *Adamts10* substitution (p.P761L) was tolerated (score = 0.33). Since SIFT did not generate a score for the *Abhd12* indel substitution, we used the PROVEAN web server (http://provean.jcvi.org) that predicts the functional effects of single and multiple amino-acid substitutions, insertions, and deletions (Choi and Chan 2015). A PROVEAN score less than −2.5 was predicted to be deleterious, whereas, a score greater than −2.5 was predicted to be neutral. The *Prx* substitution (p.R167C) was predicted to be borderline neutral (score = −2.31), whereas, the *Adamts10* substitution (p.P761L) was predicted to be neutral (score = 0.96). By contrast, the *Abhd12* indel substitution (p.L30-A32delinsS) was predicted to be functionally deleterious (score = −20.74).

**Fig. 4.**
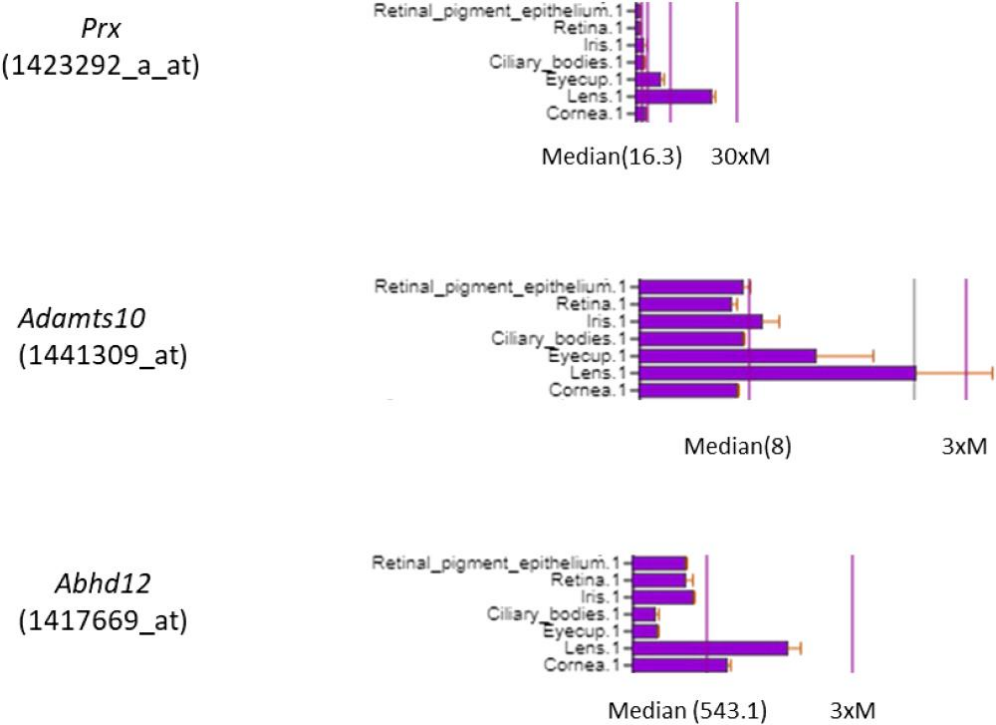
Expression levels of *Prx*, *Adamts10*, and *Abhd12* transcripts in mouse eye tissues obtained from the BioGPS gene portal. Lens expression levels indicate fold increase over median level (M) detected across all tissues.

Finally, we compared the transcript expression profile of *Prx*, *Adamts10*, and *Abhd12* in mouse eye tissues using the BioGPS gene portal (http://biogps.org) (Wu *et al*. 2016). Figure 4 shows that all three genes are expressed in the lens at higher levels than in adjacent eye tissues including the iris, ciliary body, and retina. Overall, while we cannot totally exclude *Prx* and *Adamts10*, our variant analysis data suggest that *Abhd12* was the most plausible candidate gene for *Em*.

## Discussion

The *Em* mouse inherits a spontaneous, uncharacterized, mutation underlying a reported autosomal dominant form of late-manifest (6–8 months of age), progressive, cortical cataract that has long been proposed as an animal model for age-related cataract in humans (Kuck 1990). We confirmed the cataract phenotype in commercially available homozygous *Em*/J mutant mice at 6–8 months of age but not in age-matched *Em*/J heterozygotes or CFW ancestral mice. Using whole-exome sequencing and variant analysis of *Em*/J and CFW mice, we excluded over 450 known genes for inherited and age-related forms of cataracts—with or without other ocular and/or systemic abnormalities—including those for lens crystallins, membrane and cytoskeletal proteins, DNA or RNA-binding proteins, along with many genes for syndromic/systemic forms of cataract **(**https://cat-map.wustl.edu**)**. Our variant analysis culminated in the prioritization of three genes *Prx*, *Adamts10*, and *Abhd12* each with one predicted missense variant that were not identified in any public genomic database suggesting that they were possible candidates for *Em* (Table 2). It is noteworthy that the human genes for *Adamts10* (*ADAMTS10*) and *Abhd12* (*ABHD12*) are included in commercially available panels of genes that are routinely sequenced in a clinical setting for the molecular diagnosis of inherited eye diseases including cataract and other lens disorders, anterior segment dysgenesis, and retinal degenerations (e.g. blueprintgenetics.com).

Mutations in the human gene for *Prx* (*PRX*, MIM no. 605725) on chromosome 19q13.2 have been associated with autosomal recessive Charcot Marie Tooth disease type 4F (CMT4F, MIM no. 614895) and both autosomal dominant and recessive sub-forms of Dejerine-Sottas syndrome/neuropathy (DSS/DSN, MIM no. 145900). Neither of these syndromes include cataract as a presenting clinical feature. However, *PRX* variants of uncertain significance (p.R129H, p.V1225M) have been associated with congenital cataract in humans [Yuan *et al*. 2016; Jones *et al*. 2022]. *Prx* encodes a postsynaptic density protein (PSD95), Drosophila disc large tumor suppressor (DlgA), and zonula occludens-1 protein (ZO-1) or PDZ domain protein known as periaxin that plays a key role in the maturation, packing, and hexagonal membrane organization of mouse lens fiber cells (Maddala *et al*. 2011). The *Prx* substitution (p.R167C) identified in *Em*/J mice was predicted *in silico* to have a borderline neutral/damaging effect on protein function. Further, mice lacking *Prx* did not develop cataract by 6–9 months of age and soon after they succumbed to severe co-ordination abnormalities and neuropathic pain necessitating euthanasia (Gillespie *et al*. 2000; Maddala *et al*. 2011). However, given its role in lens cell biology and its tentative association with congenital cataract in humans, we cannot exclude *Prx* as a candidate gene for *Em*.

Mutations in *ADAMTS10* (MIM no. 608990) on human chromosome 19p13.2 have been associated with Weill-Marchesani syndrome 1 (WMS1, MIM no. 277600)—a rare autosomal recessive connective tissue disorder characterized by short stature, brachydactyly, joint stiffness, occasional heart defects, and eye anomalies, including severe myopia (94%), microspherophakia (small, spherical lens, 84%), glaucoma (80%), ectopia lentis (lens displacement, 73%), and cataract (23%) (Faivre *et al*. 2003; Dagoneau *et al*. 2004). *Adamts10* encodes a secreted metalloproteinase from the ADAMTS [a disintegrin-like and metallopeptidase (reprolysis type) with thrombospondin type 1 motif, 10] superfamily of proteins with diverse roles in embryonic development and human disease (Dubail and Apte 2015; Mead and Apte 2018). However, the *Adamts10* missense substitution (p.P761L) identified in *Em*/J mice was predicted *in silico* to exert a benign effect on protein function. Whereas mice lacking or mutant for *Adamts10* have not been reported to develop WMS1-like lens defects including microspherophakia, ectopia lentis, and cataract (Mularczyk *et al*. 2018; Wang *et al*. 2019), homozygous *Em*/J mice developed cataract with full penetrance—suggesting that *Adamts10* is a marginal candidate gene for *Em*.

Mutations in *ABHD12* (MIM no. 613599) on human chromosome 20p11.21 have been associated with a rare, autosomal recessive syndrome characterized by (demyelinating) polyneuropathy, hearing loss, (cerebellar) ataxia, retinitis pigmentosa, and (early-onset) cataract (PHARC—MIM no. 612674) (Fiskerstrand *et al*. 2010; Nishiguchi *et al*. 2014; Nguyen *et al*. 2021). PHARC syndrome is a neurodegenerative disease with variable onset, severity, and progression of neurological, auditory, and ophthalmological symptoms that may confuse diagnosis with other “deaf–blind” phenotypes including Refsum disease (MIM 266500) (Fiskerstrand *et al*. 2009, 2010; Eisenberger *et al*. 2012; Yoshimura *et al*. 2015; Thimm *et al*. 2020). We note that Refsum disease is caused by mutations in the gene encoding phytanoyl-CoA hydroxylase (*PHYH*; MIM 602026), the mouse counterpart of which (*Phyh*) was excluded here (Supplementary Table 3). Typically, polyneuropathy, hearing loss, and ataxia present in the first to third decades of life; however, cases of PHARC syndrome without manifest ataxia and hearing loss have been documented (Nguyen *et al*. 2021). Retinitis pigmentosa (RP) and cataracts usually present in the second or third decades; however, the ophthalmic spectrum of PHARC syndrome includes cases with congenital cataracts and cases of “non-syndromic” RP with posterior polar cataract in the absence of other symptoms (Nishiguchi *et al*. 2014; Nguyen *et al*. 2021). While we did not observe obvious gait defects and did not test for hearing loss in *Em/*J mice, we note that studies of an *Abhd12*-related retinal phenotype are confounded since *Em*/J mice are also homozygous for the *Pde6b^rd1^* retinal degeneration mutation. In both mice and zebrafish, loss of ABHD12 function caused a PHARC-like syndrome including neurological, auditory, and retinal defects (Blankman *et al*. 2013; Tingaud-Sequeira *et al*. 2017). Whereas cataract was not reported in *Abhd12*-null mice from 5–18 months of age, inhibition of lens clarification during eye development was observed in *abhd12* knock-down zebrafish larvae (Tingaud-Sequeira *et al*. 2017). Since *Abhd12* encodes a serine hydrolase abhydrolase domain-containing protein 12 (or lysophosphatidylserine lipase ABHD12) that catalyzes the hydrolysis of 2-arachidonoyl glycerol (2-AG) to arachidonic acid and glycerol, PHARC syndrome has been classified as both an inborn error of endocannabinoid metabolism and an inborn error of phospholipid metabolism involving elevated levels of lysophosphatidylserine lipids and the endocannabinoid 2-AG (Fiskerstrand *et al*. 2010; Blankman *et al*. 2013; Lamari *et al*. 2013; Wortmann *et al*. 2015; Leishman *et al*. 2019). Unlike the variants in *Prx* and *Adamts10* above, the *Abhd12* variant (p.L30_A32delinsS) found in *Em*/J mice was predicted *in silico* to be strongly damaging at the protein function level. We note that genetic mutations in several other lipid metabolism enzymes are known to elicit cataracts in humans, including lanosterol synthase (LSS, MIM no. 600909; autosomal recessive congenital cataract, CTRCT44, MIM no. 616509) (Zhao *et al*. 2015; Zhao *et al*. 2021) and acylglycerol kinase (AGK, MIM no. 610345; Sengers syndrome, MIM no. 212350; and a non-syndromic form of autosomal recessive congenital cataract, CTRCT38, MIM no. 614691) (Aldahmesh *et al*. 2012; Mayr *et al*. 2012). Further, loss of the lipid kinase phosphatidylinositol-4-phosphate 3-kinase catalytic subunit type 2α (PI3K-C2α) leads to early senescence and cataract development in humans, mice, and zebrafish (Gulluni *et al*. 2021). Taken overall, the cross-species association with human cataract, predicted enzymatic dysfunction, and relatively strong expression in the mouse lens (Fig. 4) supports *Abhd12* as a credible candidate gene for *Em* and suggests that available *Em*/J mice may serve as an animal model for the cataract associated with human PHARC syndrome.

There are certain caveats concerning phenotype inheritance that impact this candidate gene study of *Em*/J mice. First, we were unable to map the *Em* locus to a chromosome possibly due to delayed onset and/or reduced penetrance of the reported autosomal dominant cataract inheritance (Kuck *et al*. 1981). We note that two sub-strains of *Em* mice have been reported, one with “early-cataract” onset at 5–6 months of age and the other with late-cataract onset at 8–9 months (Kuck 1990). While the 5- to 6-month-cataract sub-strain was reportedly bred to genetic homogeneity, it remains unclear that the early-onset and late-onset cataract represent homozygous and heterozygous *Em* phenotypes, respectively (Kuck 1990). Second, since mutations in *PRX*, *ADAMTS10*, and *ABHD12* are all associated with autosomal recessive syndromes in humans, we cannot rule out the possibility that *Em* exhibits autosomal recessive inheritance in mice. The *Em* phenotype arose spontaneously in an individual male (from an inbred CFW colony) that “exhibited bilateral cataracts at 11 months of age” (Kuck *et al*. 1981). Two male siblings of the affected male later developed cataract at 17 and 18 months; however one died. Progeny of the two male founders were entered into a large breeding program (∼1,000 mice) that necessitated mating of suspected cataract (i.e. pre-symptomatic) parental mice since cataract-onset occurred beyond prime breeding age. Such breeding over several years generated the *Em* sub-strain with “a continually improving yield of cataracts” and “a high probability of cataract formation” (Kuck *et al*. 1981) that may have included carriers of an autosomal recessive cataract phenotype. Finally, in the absence of firm genetic linkage data for the *Em* phenotype, we were limited to analyzing known genes for cataract/lens disorders and therefore cannot exclude the possibility that a currently unidentified gene for cataract is involved.

In conclusion, whole-exome sequencing and variant analysis has prioritized three candidate genes for *Em* that are associated with lens cell biology and human cataracts. Although we cannot formally exclude *Prx* and *Adamts10*, our data suggest that *Abhd12* is a promising candidate gene for *Em*. Further studies, including gene-editing (e.g. CRISPR-Cas9) to engineer knock-in mouse models and/or induced pluripotent stem cell (iPSC) in vitro models of lens development and cataract, will be required to confirm or exclude the causative role of all three candidate genes in generating the *Em*/J lens phenotype. Ultimately, if *Prx*, *Adamts10*, and *Abhd12* are excluded as causative genes for *Em* further variant analysis of the exome data presented here may contribute to the identification of a novel gene for cataract.

## Supplementary Material

jkad055_Supplementary_Data

## Data Availability

Supplemental Material available at figshare: https://doi.org/10.25387/g3.21861504. Supplementary Table 3 contains Em/J and CFW exome variants filtered against RefSeq genes 59, dbSNP 146, and Ensembl 106 databases. The exome datasets (FASTQ files) generated during this study are available in the Short Read Archive (SRA) repository (https://www.ncbi.nlm.nih.gov/sra/) under Accession number: PRJNA852584.
