# Supplementary material for: Whole-exome sequencing prioritizes candidate genes for hereditary cataract in the Emory mouse mutant
Source: G3 (Bethesda). 2023 Mar 9;13(5):jkad055. doi: 10.1093/g3journal/jkad055 (PMC10151407; doi:10.1093/g3journal/jkad055)

Figure S2. Allele-specific PCR amplification and gel-electrophoresis of exon 6 from *Fktn* with 3 primers (Table S1), indicated by arrows in the schematic below, confirming that the low-coverage p.S245T variant was a sequencing artefact.

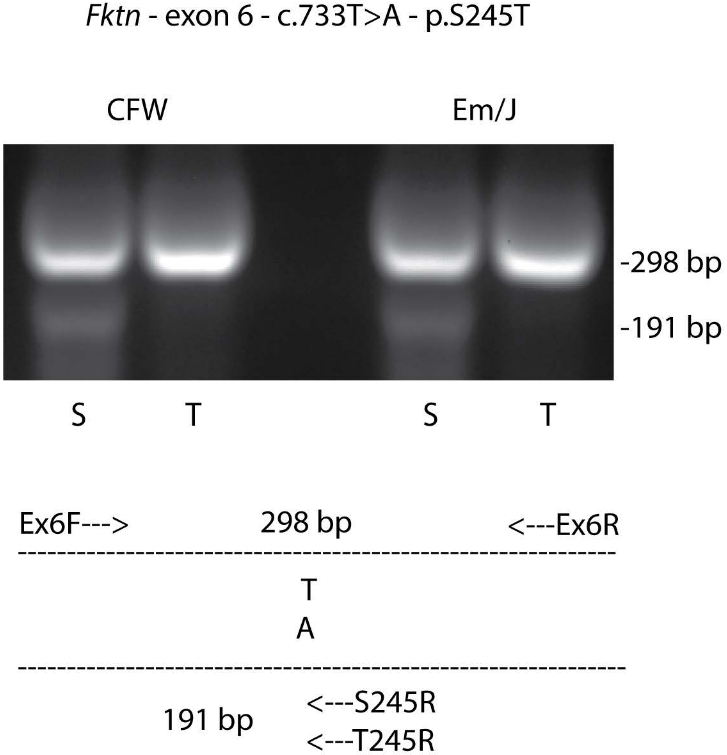

Supplement: jkad055_Supplementary_Data [file jkad055_supplementary_data.zip › Figure S2.pdf]
