# Supplementary material for: Whole-exome sequencing prioritizes candidate genes for hereditary cataract in the Emory mouse mutant
Source: G3 (Bethesda). 2023 Mar 9;13(5):jkad055. doi: 10.1093/g3journal/jkad055 (PMC10151407; doi:10.1093/g3journal/jkad055)
Supplement: jkad055_Supplementary_Data [file jkad055_supplementary_data.zip › Supplemental Material Legends.docx]

**Supplemental Material**

**Figure S1.** Representative SD-OCT scans of the anterior Em/J mouse eye at 7 months of age.

**Figure S2.** Allele-specific PCR amplification and gel-electrophoresis of exon-6 from *Fktn* with 3 primers (Table S1), indicated by arrows in the schematic below, confirming that the low-coverage p.S245T variant was a sequencing artefact.

**Table S1.** Gene-specific primers used for PCR-amplification and Sanger sequencing of novel variants in *Prx*, *Adamts10*, and *Abhd12*.

**Table S2.** Exome sequencing metrics for *Em*/J and CFW mice.

**Table S3.** *Em*/J and CFW mouse exome variants filtered against RefSeq Genes 59, dbSNP 146, and Ensembl 106 databases.
