# Supplementary figures and images for: Whole-exome sequencing prioritizes candidate genes for hereditary cataract in the Emory mouse mutant
Source: G3 (Bethesda). 2023 Mar 9;13(5):jkad055. doi: 10.1093/g3journal/jkad055 (PMC10151407; doi:10.1093/g3journal/jkad055)

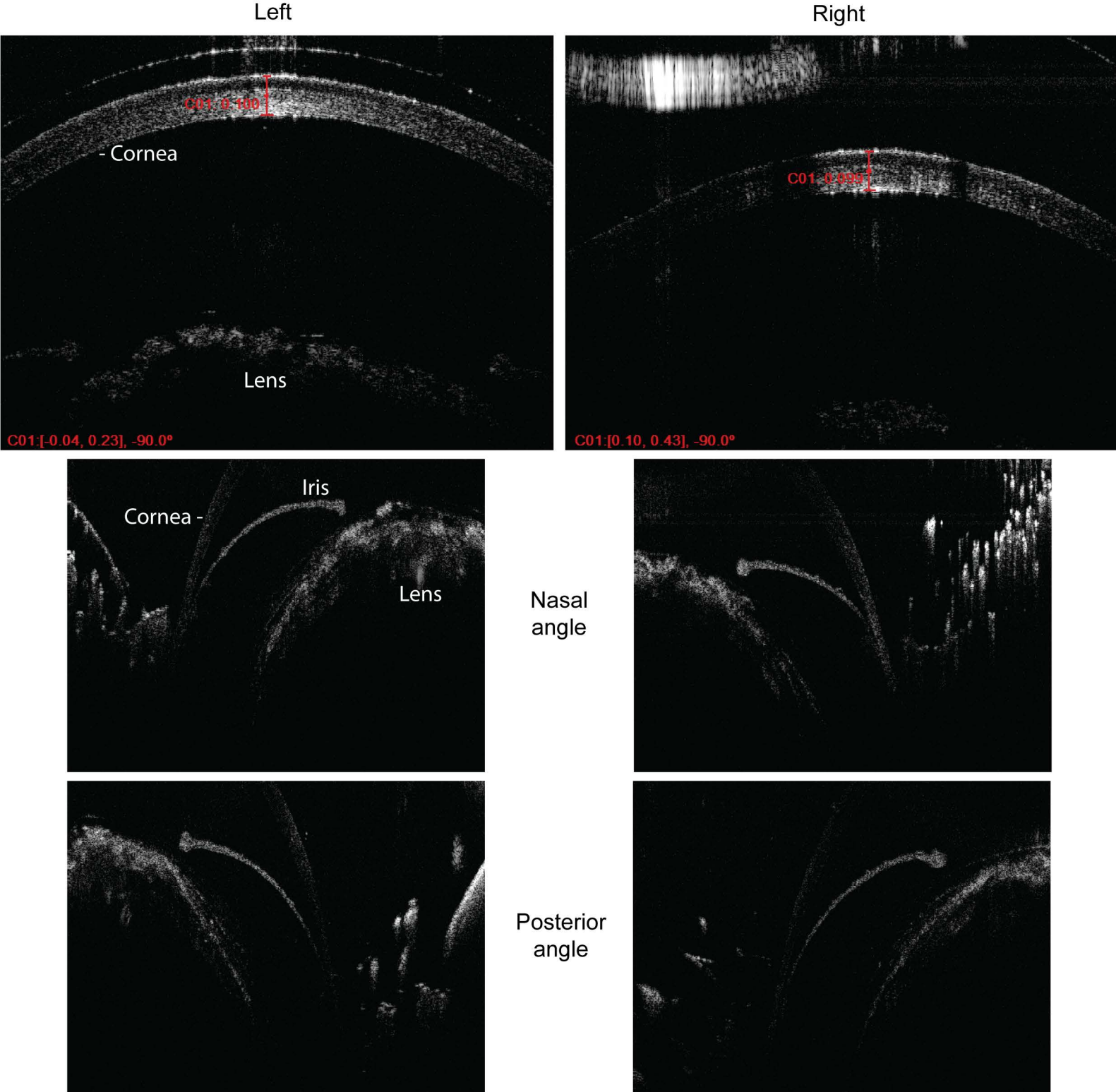

Figure S1. Representative SD-OCT scans of the anterior *Em/J* mouse eye at 7 months of age

Supplement: jkad055_Supplementary_Data [file jkad055_supplementary_data.zip › Figure S1.pdf]
